# Appraisal the significance of completion hysterectomy after definitive concurrent chemoradiotherapy for patients with locally advanced cervical adenocarcinoma: the ATTRACT study

**DOI:** 10.1097/JS9.0000000000003549

**Published:** 2025-11-05

**Authors:** Xi-Lin Yang, Jia-Wei Zhu, Yun-Can Zhou, Yan You, Hui Guan, Zheng Miao, Peng Peng, Yong-Lan He, Qiu Guan, Zhi-Wei Yang, Yue Zhang, Jun-Fang Yan, Ke Hu, Fu-Quan Zhang

**Affiliations:** a Department of Radiation Oncology, Peking Union Medical College Hospital, Chinese Academy of Medical Sciences & Peking Union Medical College, Beijing, People’s Republic of China; b Department of Pathology, Peking Union Medical College Hospital, Chinese Academy of Medical Sciences & Peking Union Medical College, Beijing, People’s Republic of China; c Department of Gynecology, Peking Union Medical College Hospital, Chinese Academy of Medical Sciences & Peking Union Medical College, Beijing, People’s Republic of China; d Department of Radiology, Peking Union Medical College Hospital, Chinese Academy of Medical Sciences & Peking Union Medical College, Beijing, Republic of China; e Department of Radiation Oncology, State Key Laboratory of Complex Severe and Rare Diseases, Peking Union Medical College Hospital, Chinese Academy of Medical Sciences & Peking Union Medical College, Beijing, People’s Republic of China

**Keywords:** adenocarcinoma, cervical cancer, chemoradiotherapy, hysterectomy, survival

## Abstract

**Objective:**

To appraise whether the completion hysterectomy after concurrent chemoradiotherapy (CCRT) would improve the survival outcomes for patients with locally advanced cervical adenocarcinoma (LACA).

**Methods:**

This study was conducted based on a large cohort including more than 200 LACA patients from Peking Union Medical College Hospital. The included patients were divided into the CCRT alone and CCRT + surgery groups, where overall survival (OS), progression-free survival (PFS), loco-regional-free survival (LRFS), and distant metastasis-free survival (DMFS) were compared between the two groups before and after propensity scoring matching (PSM). Cox regression analysis was performed in the CCRT alone group to identify the risk factors impairing the survival probability. The survival outcomes were further compared between the CCRT alone and the CCRT + surgery group in different risk subgroups identified from Cox regression analysis.

**Results:**

CCRT + surgery was observed to outperform CCRT alone in OS, PFS, LRFS, and DMFS before PSM (all *P* < 0.05), while the benefit of surgery could not be maintained after PSM except for DMFS (3-year DMFS: 76.8 vs. 60.2%, *P* = 0.035). Uterus involvement (UI) was identified as the only risk factor for the CCRT alone group. Surgery was found to increase the survival probability for patients with UI (all *P* < 0.05), while it did not bring additional survival benefit for patients without UI (all *P* > 0.05). In the CCRT + surgery group, patients with pathological residual disease (RD) ≥ 1/2 myometrial infiltration (MI) had significantly decreased survival compared to patients with RD<1/2MI or patients without RD (all *P* < 0.05). Furthermore, postoperative chemotherapy didn’t improve survival outcomes in patients with RD<1/2MI, while it seemed to bring additional benefit for patients with RD≥1/2MI in terms of PFS with marginal significance (*P* = 0.055).

**Conclusion:**

Completion hysterectomy after CCRT could increase DMFS for LACA patients, while not every LACA patient would retain OS benefit from surgery, except for those with positive UI. Survival differences existed in different degrees of RD among patients receiving post-radiation surgery and postoperative chemotherapy, demonstrating a trend toward PFS benefit for patients with RD≥1/2MI.

**HIGHLIGHTS**

- Completion hysterectomy after CCRT could increase DMFS for LACA patients.
- Completion hysterectomy after CCRT could improve the survival outcomes for patients with UI, not for those without UI.
- Patients with pathological RD≥1/2MI had significantly decreased survival probability compared to patients without RD and with RD<1/2MI.
- Postoperative chemo might increase the PFS for patients with RD≥1/2MI with marginal significance.

## Introduction

Cervical cancer was one of the most important culprits undermining women’s health globally, and it was estimated that approximately 661 021 new cases and 348 189 related deaths worldwide would occur in 2022^[1]^. Generally, the incidence of squamous cell carcinoma of cervix (SCC) has decreased over time due to the wide introduction of human papillomavirus (HPV) vaccination in the whole world, on the contrary, the incidence of adenocarcinoma of cervix (AC) has steadily increased given that more than 15% of AC was not HPV related, which was difficult to be prevented solely relying on HPV vaccination^[2–4]^. Hence, AC was further divided into HPV associated (HPVA) and HPV independent (HPVI) types with distinct survival outcomes according to the latest World Health Organization categorization in 2020^[5–8]^.

Previous researches have disclosed the dire outcome of AC patients compared to SCC patients, whether in early stage or locally advanced stage^[9,10]^; however, the treatment modality for AC has not been modified in the newly released National Comprehensive Cancer Network (NCCN) guideline, which maintains the same treatment principles for both AC and SCC patients^[11]^. In specific, concurrent chemoradiotherapy (CCRT), consisting of external beam radiotherapy (EBRT) and intracavity brachytherapy (BRT) concurrently with chemotherapy, was the standard treatment for locally advanced cervical cancer (LACC). Growing evidence has indicated that AC might be more radio-resistant than SCC, leading to residual disease (RD) existing in 40–50% of AC patients after CCRT^[12–14]^, which calls for reappraisal of the significance of post-radiation surgery in AC patients. The post-radiation surgery was listed as category 3 recommendation in the latest NCCN guideline, resulting from that the surgery did not bring additional survival benefits for SCC patients after definitive CCRT^[15,16]^. A few historical researches have assessed the role of completion hysterectomy after CCRT in AC patients with conflicting results, where the Brazilian study, including 80 AC patients, demonstrated that surgery did not benefit LACA patients^[17]^, while Yang *et al* reached the conclusion that completion hysterectomy was associated with improving survival^[18]^.

Therefore, reappraisal the status of completion hysterectomy after definitive CCRT in locally advanced cervical adenocarcinoma (LACA) patients was necessary in the era with more detailed classification of AC patients. Besides, patients with obvious RD confirmed by gynecological examination and pelvic magnetic resonance imaging (MRI) would not be incorporated into the analysis in this study, given that the beneficial role of completion hysterectomy for AC patients with obvious RD has been established by previous studies^[18–20]^. We would assess if completion hysterectomy would bring additional benefit for LACA patients without obvious RD after CCRT in the current study.

## Materials and methods

### The ATTRACT study

The ATTRACT (ev**a**luation of **t**he **t**reatment **r**esponse and prognosis of cervical **a**deno**c**arcinoma after radio**t**herapy) study was an ongoing large retrospective cohort study evaluating the treatment response and prognosis of AC patients receiving definitive or adjuvant radiotherapy based on the prestigious status of our hospital in treating gynecological cancer, which resulted in the nationwide convergence of rare gynecological cancer patients, including patients with AC. The ethical approval for this study has been obtained from the Institutional Review Board (IRB) of our hospital. Also, written informed consents were acquired from every participant. Notably, we registered the current study in http://clinicaltrials.gov.

### Study participants

The LACA patients undergoing definitive CCRT from the ATTRACT database were selected, and those with Karnofsky performance score ≥ 80 and aged more than 18 years old were included in the analysis. The exclusion criteria were: (1) patients with obvious RD 3 months after the completion of CCRT confirmed by gynecological examination and MRI; (2) patients with primary diagnosis of stage IV disease; (3) patients did not receive concurrent chemotherapy during the period of radiotherapy; (4) patients died from surgical complications (died within 1 month after surgery); and (5) patients with incomplete follow-up information. In addition, all the patients diagnosed before the release of the 2018 International Federation of Gynecology and Obstetrics (FIGO) staging system would be assessed and restaged based on the 2018 FIGO staging system. Furthermore, every corresponding pathological specimen belonging to the included patients would be reviewed again by the pathologist who specializes in gynecological oncology to determine the HPVA or HPVI type.

### Treatment

Every included patient received definitive CCRT including EBRT and BRT concurrent with platinum-based chemotherapy prescribed weekly with single-agent or triweekly with double-agent. Specifically, the volumetric modulated arc therapy (VMAT) or other techniques, including intensity modulated radiotherapy and tomotherapy, were employed during the EBRT session. Accordingly, uterine body, major ligaments, uterine adnexa, parametrial space, and parts of vagina would be included in the radiation region. Besides, lymph node drainage basin, including the common iliac, external iliac, presacral, internal iliac, obturator, and retroperitoneal, when lymph node metastasis was spotted in this region, would also be covered in the clinical target volume (CTV). Notably, the retroperitoneal region would also be covered when extensive pelvic lymph nodes present, according to the physicians’ decision. A dosage of 50.4 Gy/28 f and 60.2 Gy/28 f would be prescribed to the CTV and positive lymph nodes or suspicious metastatic lymph nodes, respectively. After the completion of EBRT, BRT with a dosage of 30 Gy/5 f would be performed for every patient through a two-dimensional (2D) or three-dimensional (3D) technique. Of note, interstitial BRT would be categorized as a 3D technique.

### Cohort definition

Patients would receive a complete set of follow-up, including chest and abdominal CT, pelvic MRI, gynecological examination, and blood test 1 and 3 months after the completion of primary treatment. Patients with obvious RD 3 months after primary treatment would be treated with completion hysterectomy and excluded from the current study, while patients without obvious RD would either accept completion hysterectomy or close surveillance according to patients’ preference and physicians’ decision. Normally, laparoscopic surgery would be performed for those receiving post-radiation surgery, given that patients without obvious RD were included in the current study. Besides, postoperative chemotherapy with TP (paclitaxel and cisplatin) regimen for four cycles would be prescribed based on the pathological findings. Therefore, patients without obvious RD would be assigned to CCRT alone and CCRT + surgery group depending on whether they have chosen completion hysterectomy or not. Besides, the physicians would decide if the postoperative chemotherapy should be prescribed according to their experience. Additionally, information on age, body mass index (BMI), FIGO stage (stage I, II, III), pathology (HPVA, HPVI, or Unknown), tumor size, radiation duration, uterus involvement (UI) (Yes or No), EBRT technique (VMAT or Other), BRT technique (2D or 3D), neoadjuvant chemotherapy (Yes or No), adjuvant chemotherapy (Yes or No), pretreatment, and aftertreatment CA125 concentration would be collected. Notably, the presence UI was diagnosed based on pretreatment MRI and every corresponding MRI scanning reports would be reviewed and the reports filed by senior radiologist who majored in gynecological oncology would be accepted or the image would be reviewed again by the experienced radiologist from our team and patients with disease spreading to any part of uterine corpus including myometrium and endometrium would be identified as UI. Besides, adjuvant chemotherapy was defined as chemotherapy delivered after CCRT in the CCRT alone group and chemotherapy after CCRT but before surgery in the CCRT + surgery group. Moreover, the aftertreatment CA125 concentration would be collected 3 months after CCRT or before surgery.

### Outcomes

The primary endpoint in this study was overall survival (OS), defined as the duration from diagnosis to the date of death or last follow-up. The second endpoints were progression-free survival (PFS), loco-regional-free survival (LRFS), and distant metastasis-free survival (DMFS), which were estimated as the time from treatment to the date of any sign of progression, loco-regional recurrence, and newly found distant metastasis, respectively. The OS, PFS, LRFS, and DMFS were compared between the CCRT alone and the CCRT + surgery group before and after propensity scoring matching (PSM), which was usually employed to alleviate the imbalance of the baseline characteristics across groups^[21]^. Of note, variables showing significant imbalance (*P* < 0.1) between two groups in preliminary analyses were prioritized for inclusion and nearest neighbor 1:1 matching with a caliper of 0.05 was applied, ensuring matches were within a close propensity score range.

### Statistical analysis

Categorical variables were displayed as percentages or frequencies and compared using the Pearson *χ*^2^ test, while continuous variables were listed as means with standard deviation and compared with the *t-*test. Kaplan–Meier curves were plotted, and the differences in terms of survival outcomes between the CCRT alone and the CCRT + surgery group were compared using the log-rank test. Subsequently, univariate and multivariate Cox regression analysis were conducted in the CCRT alone group to identify the independent risk factors affecting the survival outcomes for these patients. Accordingly, the survival outcomes were compared between CCRT and CCRT + surgery in different risk subgroups. Moreover, the significance of the degree of pathological RD in CCRT + surgery was investigated. In specific, patients in the CCRT + surgery group were divided into no RD, with RD<1/2 myometrial infiltration (MI), and with RD≥1/2MI subgroups according to pathological findings. Accordingly, the survival outcomes were compared among three subgroups. Furthermore, the survival differences between adding or not adding postoperative chemotherapy would be separately compared within each of the three subgroups derived from the pathological findings.

All analyses were performed using SPSS v24.0 and R software (version 3.6.1; http://www.r-project.org). A two-tailed *P* < 0.05 was considered statistically significant. And the work has been reported in line with the STROCSS criteria^[22]^.

## Results

### Patients characteristics

**Figure 1.**
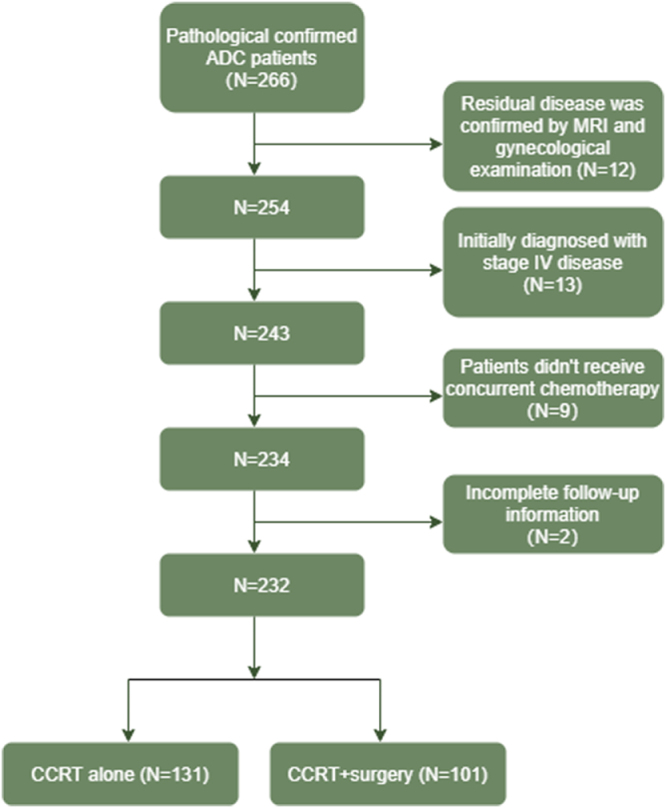
The detailed flow chart depicts the selection process of the patients.

Collectively, 232 LACA patients receiving CCRT as primary treatment were included in the final analysis, with 131 and 101 patients assigned to the CCRT alone and the CCRT + surgery group, respectively. Relatively safe surgical operations have been delivered for patients in CCRT + surgery group without patients died within 1 month after surgery. And the selection process was listed in detail in Figure 1. Patients with younger age (50.21 ± 10.06 vs. 57.44 ± 11.82, *P* < 0.001), receiving VMAT during EBRT session (89.1 vs. 79.4%, *P* = 0.047), 2D BRT (81.2 vs. 64.1%, *P* = 0.004), and adjuvant chemotherapy (42.6 vs. 17.6%, *P* < 0.001) tended to favor post-radiation surgery. Besides, over 90% of patients received single-agent concurrent chemotherapy and no significant difference was observed between the two groups (91.6 vs. 91.1%; *P* = 0.907) (Table 1). Furthermore, age, pathology, tumor size, neoadjuvant chemotherapy, adjuvant chemotherapy, EBRT technique, BRT technique, and aftertreatment CA125 concentration were included for PSM analysis, resulting in the balance of baseline characteristics between CCRT alone and CCRT + surgery group (all *P* > 0.05) (Table 1).

**Table 1:** Comparison of clinical and pathological characteristics between the CCRT alone group and the CCRT + surgery group before and after PSM

Variable	Before PSM				After PSM			
	CCRT alone (*N* = 131)	CCRT + surgery (*N* = 101)	*P*-value	SMD	CCRT alone (*N* = 50)	CCRT + surgery (*N* = 50)	*P*-value	SMD
Age	57.44 ± 11.82	50.21 ± 10.06	**<0.001**	0.718	53.02 ± 9.99	52.10 ± 10.86	0.660	0.085
BMI	23.91 ± 3.58	24.22 ± 3.86	0.527	0.081	24.58 ± 3.94	24.42 ± 3.93	0.836	0.042
FIGO stage, *n* (%)			0.608	0.132			1.000	0.040
I	11 (8.40)	9 (8.91)			4 (8.00)	5 (10.00)		
II	63 (48.09)	42 (41.58)			23 (46.00)	23 (46.00)		
III	57 (43.51)	50 (49.50)			23 (46.00)	22 (44.00)		
Pathology, *n* (%)			**0.003**	0.282			0.748	0.042
HPVA	82 (62.60)	49 (48.51)			31 (62.00)	28 (56.00)		
HPVI	43 (32.82)	34 (33.66)			16 (32.00)	17 (34.00)		
Unknown	6 (4.58)	18 (17.82)			3 (6.00)	5 (10.00)		
Tumor size	3.86 ± 1.69	4.22 ± 1.54	0.097	0.233	3.92 ± 1.62	4.01 ± 1.56	0.767	0.061
Radiation duration	56.25 ± 11.99	57.59 ± 11.83	0.403	0.133	55.70 ± 7.15	55.48 ± 7.17	0.878	0.031
Neoadjuvant, *n* (%)			0.099	0.202			0.806	0.048
No	110 (83.97)	76 (75.25)			40 (80.00)	39 (78.00)		
Yes	21 (16.03)	25 (24.75)			10 (20.00)	11 (22.00)		
Concurrent regimen			0.907	0.064			0.577	0.041
Single agent	120 (91.60)	92 (91.09)			45 (90.00)	46 (92.00)		
Double agent	11 (8.39)	9 (8.91)			5 (10.00)	4 (8.00)		
Concurrent cycle								
Single agent	5.12 ± 1.35	5.07 ± 1.44	0.802	0.120	5.07 ± 1.31	5.09 ± 1.29	0.819	0.029
Double agent	3.08 ± 1.22	3.13 ± 1.57	0.766	0.223	3.11 ± 1.59	3.09 ± 1.42	0.835	0.047
Adjuvant, *n* (%)			**<0.001**	0.506			0.680	0.083
No	108 (82.44)	58 (57.43)			30 (60.00)	32 (64.00)		
Yes	23 (17.56)	43 (42.57)			20 (40.00)	18 (36.00)		
UI, *n* (%)			0.400	0.111			0.838	0.041
No	76 (58.02)	53 (52.48)			31 (62.00)	30 (60.00)		
Yes	55 (41.98)	48 (47.52)			19 (38.00)	20 (40.00)		
EBRT tech, *n* (%)			**0.047**	0.312			1.000	0.074
VAMT	104 (79.39)	90 (89.11)			47 (94.00)	46 (92.00)		
Other	27 (20.61)	11 (10.89)			3 (6.00)	4 (8.00)		
BRT tech, *n* (%)			**0.004**	0.437			0.280	0.098
2-Dimensional	84 (64.12)	82 (81.19)			32 (64.00)	37 (74.00)		
3-Dimensional	47 (35.88)	19 (18.81)			18 (36.00)	13 (26.00)		
Pretreatment CA125	88.66 ± 396.42	61.79 ± 94.59	0.517	0.284	46.88 ± 71.58	49.06 ± 62.34	0.872	0.035
aAftertreatment CA125	24.22 ± 29.69	16.80 ± 18.38	**0.027**	0.404	18.02 ± 18.90	17.95 ± 24.25	0.988	0.003

BMI, body mass index; BRT, brachytherapy; CCRT, concurrent chemoradiotherapy; HPVA, HPV associated; HPVI, HPV independent; PSM, propensity scoring matching; UI, uterus involvement; VMAT, volumetric modulated arc therapy.

^a^ Aftertreatment CA125: CA125 concentration 3 months after CCRT or before surgery.

Bold values stand for *P*<0.05

### Survival outcomes

**Figure 2.**
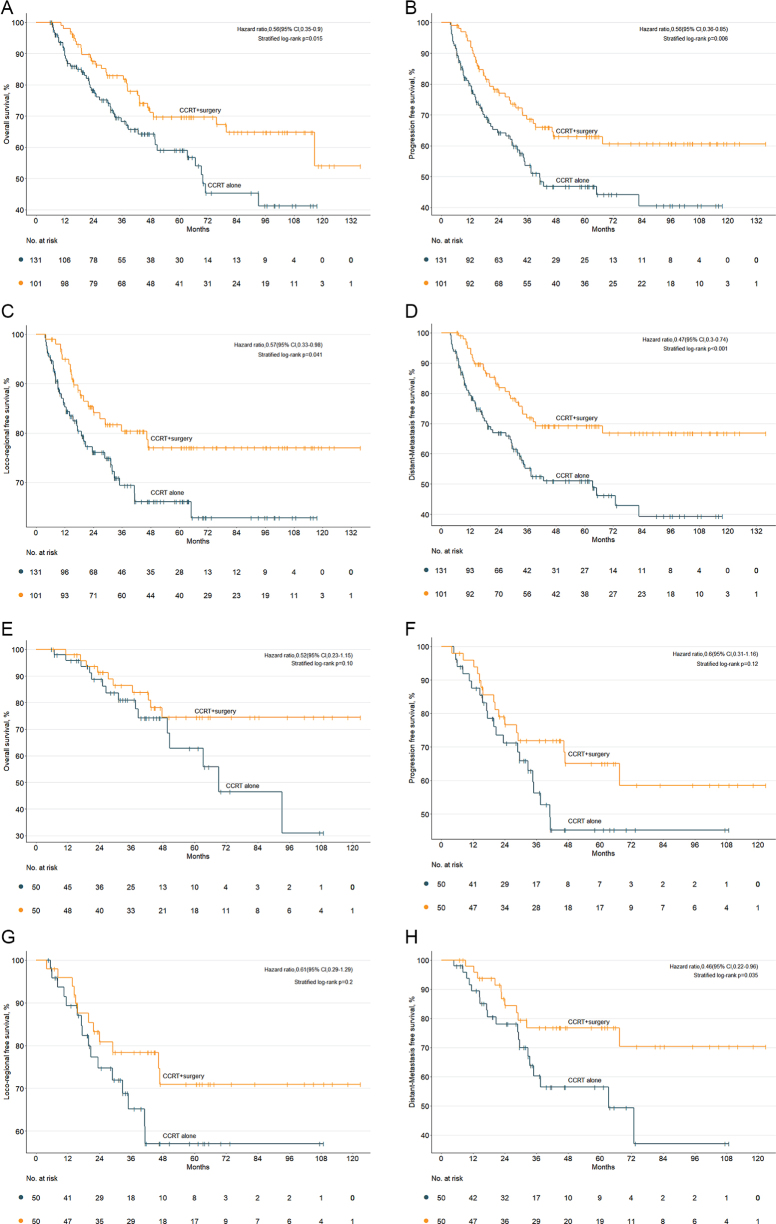
The comparison of OS (A), PFS (B), LRFS (C), and DMFS (D) between CCRT alone and CCRT + surgery group before the propensity scoring matching (PSM). The comparison of OS (E), PFS (F), LRFS (G), and DMFS (H) between the CCRT alone and the CCRT + surgery group after PSM.

Comparatively better 3-year OS (71.9%, 95% CI: 64.2–79.7 vs. 53.6%, 95% CI: 48.7–58.5; *P* = 0.015), 3-year PFS (81.7%, 95% CI: 77.8–85.6 vs. 68.2%, 95% CI: 63.7–72.7; *P* = 0.006), 3-year LRFS (80.3%, 95% CI: 76.2–84.4 vs. 69.3%, 95% CI: 64.7–73.9; *P* = 0.041), and 3-year DMFS (71.9%, 95% CI: 76.6–67.2 vs. 55.2%, 95% CI: 50.3–60.1; *P* < 0.001) were observed in CCRT + surgery group compared to CCRT alone group before PSM (Fig. 2a–d). However, the superiority over CCRT alone group could only be maintained in terms of 3-year DMFS (76.8%, 95% CI: 70.4–83.2 vs. 60.2%, 95% CI: 52.4–68.0; *P* = 0.035) after the adjustment of PSM (Fig. 2h). Comparable 3-year OS, PFS, and LRFS were observed between CCRT alone and CCRT + surgery group after PSM (all *P* > 0.05) (Fig. 2e-g), which should be interpretation with caution due to the small sample size of two groups (*N* =50 in each group), resulting in less efficient statistical power (Supplemental Digital Content Table S1, available at: http://links.lww.com/JS9/F438).

### Risk factors for patients in the CCRT alone group

**Figure 3.**
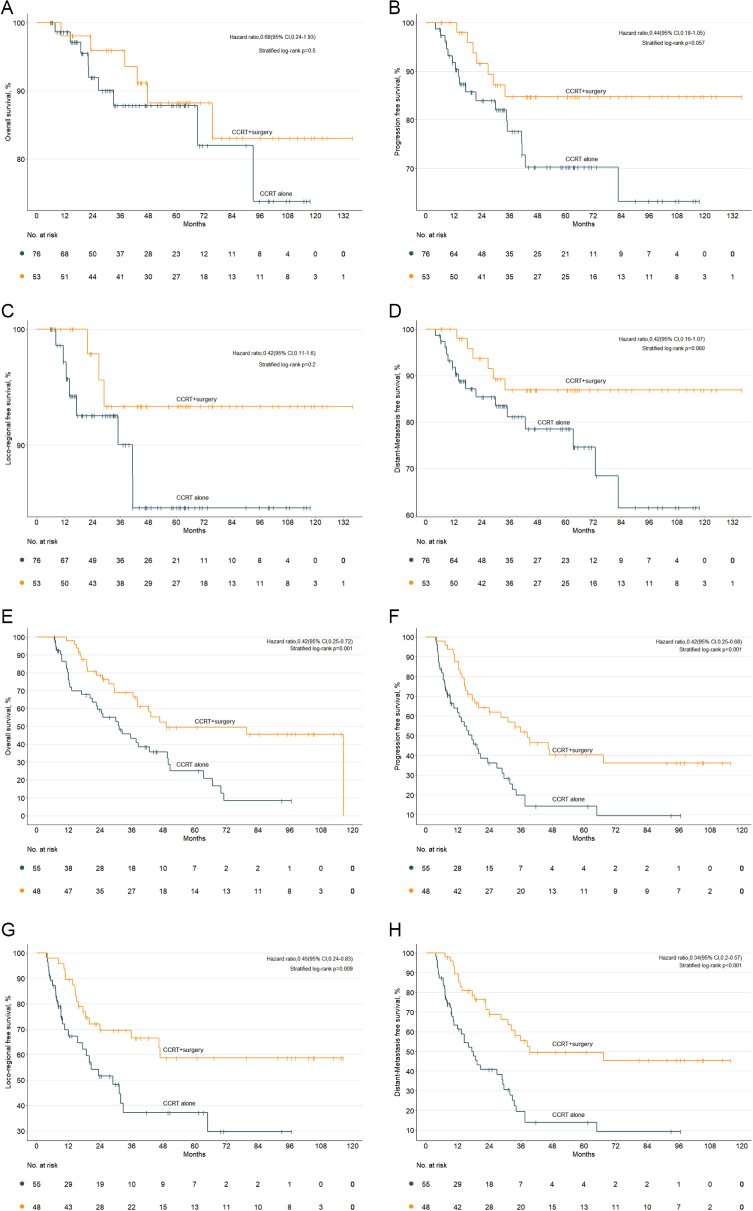
The comparison of OS (A), PFS (B), LRFS (C), and DMFS (D) between CCRT alone and CCRT + surgery group among patients without uterus involvement. The comparison of OS (E), PFS (F), LRFS (G), and DMFS (H) between the CCRT alone and the CCRT + surgery group among patients with uterus involvement.

Univariate Cox regression was performed in the CCRT alone group. As a result, FIGO stage, pathology, tumor size, and UI were found to be associated with the survival outcomes for these patients, while UI was the only risk factor maintained in the multivariate Cox regression analysis (Table 2). Therefore, survival outcomes of patients receiving CCRT alone and CCRT + surgery were separately compared among patients with UI or not. In detail, CCRT + surgery did not outperform CCRT alone in 3-year OS (93.5%, 95% CI: 90.0–97.0 vs. 87.8%, 95% CI: 83.5–92.1; *P* = 0.5), 3-year PFS (84.7%, 95% CI: 79.5–89.9 vs. 77.6%, 95% CI: 72.3–82.9; *P* = 0.057), 3-year LRFS (93.3%, 95% CI: 89.7–96.9 vs. 90.0%, 95% CI: 86.1–93.9; *P* = 0.2), and 3-year DMFS (86.9%, 95% CI: 82.0–91.8 vs. 81.1%, 95% CI: 76.2–86.0; *P* = 0.060) among patients without UI (Fig. 3a–d). Intriguingly, patients receiving CCRT + surgery had better outcomes than those undergoing CCRT alone among patients with UI in terms of 3-year OS (66.4%, 95% CI: 59.3–73.5 vs. 40.9%, 95% CI: 33.7–48.1; *P* = 0.001), 3-year PFS (51.8%, 95% CI: 44.5–59.2 vs. 19.8%, 95% CI: 13.6–26.0; *P* < 0.001), 3-year LRFS (66.5%, 95% CI: 59.4–73.6 vs. 37.1%, 95% CI: 29.0–45.2; *P* = 0.009), and 3-year DMFS (55.4%, 95% CI: 47.8–63.0 vs. 19.5%, 95% CI: 13.3–25.7; *P* < 0.001) (Fig. 3e–h). In conclusion, post-radiation surgery could bring additional benefit for patients with UI, while patients without UI could not benefit from surgery.

**Table 2:** Univariate and multivariate Cox regression analysis on risk factors of overall survival in CCRT alone cohort (*N* = 131)

	UUnivariate analysis			MMultivariate analysis	
Variable	H*R*	95% CI	*P-*value	HR	95% CI	*P*-value
**Age**	1.00	(0.97–1.02)	0.874			
**BMI**	0.96	(0.88–1.05)	0.350			
**FIGO stage**						
I	ref					
II	5.23	(0.70–39.06)	0.107			
III	9.46	(1.27–70.51)	**0.028**			
**Pathology**						
HPVA	ref					
HPVI	4.84	(1.63–14.42)	**0.002**			
Unknown	2.56	(1.40–4.70)	**0.005**			
**Tumor size**	1.26	(1.06–1.50)	**0.008**			
**Radiation duration**	1.01	(0.98–1.04)	0.599			
**Neoadjuvant**						
No	ref					
Yes	1.08	(0.48–2.42)	0.857			
**Concurrent regimen**						
Single agent	ref					
Double agent	1.27	(0.59–1.90)	0.741			
**Adjuvant**						
No	ref					
Yes	0.98	(0.72–1.39)	0.129			
**UI**						
No	ref			ref		
Yes	8.80	(4.20–18.43)	**<0.001**	7.75	(3.57–16.85)	**<0.001**
**EBRT tech**						
VAMT	ref					
Other	1.49	(0.76–2.95)	0.281			
**BRT tech**						
2-Dimensional	ref					
3-Dimensional	1.60	(0.83–3.09)	0.161			
**Pretreatment CA125**	1.00	(1.00–1.00)	0.148			
**^a^****Aftertreatment CA125**	1.02	(0.98–1.11)	0.227			

BMI, body mass index; BRT, brachytherapy; CCRT, concurrent chemoradiotherapy; CI, confidence interval; HPVA, HPV associated; HPVI, HPV independent; HR, hazard ratio; PSM, propensity scoring matching; UI, uterus involvement; VMAT, volumetric modulated arc therapy.

^a^ Aftertreatment CA125: CA125 concentration 3 months after CCRT or before surgery.

Bold values stand for *P*<0.05

### Survival differences among patients receiving CCRT + surgery

According to pathological findings, patients receiving CCRT + surgery were divided into three subgroups, namely, patients without RD, patients with RD<1/2MI, and patients with RD≥1/2MI. Comparable survival outcomes were observed between patients without RD and patients with RD<1/2MI regarding 3-year OS (87.7%, 95% CI: 79.6–95.8 vs. 88.2% 95% CI: 82.7–93.7; *P* = 0.721), 3-year PFS (89.4%, 95% CI: 83.8–94.7 vs. 85.7%, 95% CI: 79.9–91.5; *P* = 0.557), 3-year LRFS (95.0%, 95% CI: 90.2–99.8 vs. 95.2%, 95% CI: 90.7–99.7; *P* = 0.822), and 3-year DMFS (92.3%, 95% CI: 85.0–99.6 vs. 85.7%, 95% CI: 77.9–91.5; *P* = 0.081). However, significant decreased 3-year OS (60.1%; 95% CI: 51.2–69.0), 3-year PFS (43.5%; 95% CI: 34.4–52.6), 3-year LRFS (53.5%; 95% CI: 44.5–62.5), and 3-year DMFS (49.8%; 95% CI: 40.2–59.4) could be observed in patients with RD≥1/2MI when compared to patients without RD and with RD<1/2MI (all *P* < 0.05) (Supplemental Digital Content Fig. S1a–d, available at: http://links.lww.com/JS9/F438).

The role of postoperative chemotherapy was further assessed in different subgroups with different degrees of RD among patients receiving CCRT + surgery. Patients without RD would not receive further treatment in our institution therefore, the significance of postoperative chemotherapy among these patients was not assessable in this study. In addition, patients with RD<1/2MI could not benefit from postoperative chemotherapy (Supplemental Digital Content Fig. S2a–d, available at: http://links.lww.com/JS9/F438) while patients with RD≥1/2MI were inclined to benefit from postoperative chemotherapy in terms of PFS (57.6%, 95% CI: 45.3–70.0 vs. 24.5%, 95% CI: 12.6–36.4) (*P* = 0.055) (Supplemental Digital Content Fig. S2f, available at: http://links.lww.com/JS9/F438) and LRFS (69.9%, 95% CI: 58.8–81.0 vs. 32.7%, 95% CI: 19.9–45.5) (*P* = 0.069) with marginal significance (Supplemental Digital Content Fig. S2e, available at: http://links.lww.com/JS9/F438). However, additional benefit for OS (*P* = 0.3) (Supplemental Digital Content Fig. S2e, available at: http://links.lww.com/JS9/F438) and DMFS (*P* = 0.13) (Supplemental Digital Content Fig. S2h, available at: http://links.lww.com/JS9/F438) could not be further achieved from postoperative chemotherapy for those with RD≥1/2MI, which should be further validated with bigger sample size in the future given the relatively low statistical power (Supplemental Digital Content Table S1, available at: http://links.lww.com/JS9/F438).

## Discussion

### Summary of main results

The completion hysterectomy was found to benefit LACA patients without obvious RD after definitive CCRT in terms of DMFS. Subsequently, we performed Cox regression analysis in patients receiving CCRT alone in order to find who might benefit from additional treatment, which demonstrated that UI was the only risk factor. The survival differences between CCRT alone and CCRT + surgery were separately compared in patients with UI and without UI, which revealed that surgery could improve the survival outcomes for patients with UI, while patients without UI could not gain additional benefit from surgery. Furthermore, patients with pathological RD≥1/2MI had significantly decreased survival probability compared to patients without pathological RD and with RD<1/2MI among patients receiving CCRT + surgery. Additionally, patients with RD<1/2MI could not benefit from postoperative chemotherapy, which however might increase the PFS for patients with RD≥1/2MI with marginal significance.

### Results in the context of published literature

The completion hysterectomy after definitive CCRT has a long history of being appraised for LACC, which did not bring additional benefit for SCC patients while always accompanied by incompatible surgical complications leading to the decreased quality of life for these patients^[16,19,20,23]^. Nevertheless, the post-radiation surgery might possess great potential in benefiting AC patients after CCRT, given that AC was more resistant to radiotherapy than SCC, therefore leading to a bigger chance of RD^[13,14]^. Previous studies have assessed the role of completion surgery for LACA patients after CCRT with conflicting results^[13,17,18]^. Notably, baseline imbalance was not adjusted in existing studies due to the small sample size, which to some degree impairs the credibility and universality of these studies. With a larger sample size in the current study, we performed PSM to balance the baseline characteristics with 50 patients left in each group. Intriguingly, post-radiation surgery improved DMFS other than LRFS for these patients, and the benefit of DMFS did not translate into the improvement of OS either. A plausible explanation would be that the LRFS of LACA patients was primarily contributed by radiation, while limited benefit could be obtained from post-radiation surgery. However, surgical resection of the primary tumor could reduce tumor burden, thereby decreasing sources of subsequent distant dissemination^[24]^. Besides, AC demonstrated a higher propensity for early micro-metastasis, which could be better managed by total removal of the tumor than local radiotherapy^[25]^, which partially explained the beneficial role of surgery for DMFS among LACA patients.

As to the identification of patients who might benefit from additional surgery, we performed Cox regression analysis exclusively in patients undergoing CCRT alone to identify the risk factors, which was consistent with the concept from Coate who suggested that untreated or less treated patients were the optimal population to determine risk factors^[26]^. Eventually, the presence of UI stood out to be the only risk factor for patients in the CCRT alone group and surgery was firmly established as the approach to improve survival outcomes among patients with UI, while patients without UI could not benefit more from post-radiation surgery. A potential explanation would be that over 70% of the patients in this study accepted 2D BRT treatment, which could also cover the whole uterine cavity, while failed to satisfy the higher dosage covering of the uterine lesion due to the difficulty in adjusting dosage distribution according to the specific location and size of the lesion^[27]^. Thereby, pathological RD could be revealed in a larger proportion of patients with UI than those without, which could be further verified in the current study, with 81.3 and 58.5% pathological RD were observed in patients with UI and those without, respectively.

Moreover, the significance of the degree of pathological RD was further investigated in patients receiving CCRT + surgery and pathological RD≥1/2MI was clearly an important factor compromising the survival outcomes for these patients, which was consistent with previous research where the pathological RD was identified as an independent risk factor for LACA patients receiving CCRT and post-radiation surgery^[18]^. However, the survival difference between different degrees of RD was not further compared in the previous study. Patients without RD and with RD<1/2MI had comparable survival probability in our study, which highlighted the heterogeneity among patients with pathological RD, and the degree of pathological RD was pivotal in discerning patients with poor outcomes from those with relatively better outcomes. Additionally, postoperative could not further improve the survival outcomes for patients with pathological RD<1/2MI, while a trend of increased PFS could be observed for patients with RD≥1/2MI, which might need further validation in future studies with a larger sample size.

### Strengths and weaknesses

Although previous studies have appraised the significance of completion hysterectomy after CCRT for LACA patients, this study might outperform previous studies from three perspectives. First, the baseline characteristics were adjusted with the PSM method, which further increased the persuasiveness of the result. Indeed, we did not observe OS advantages obtained from additional surgery after CCRT, like previous studies did. Second, specific LACA patients who might benefit from completion surgery after CCRT were identified, which aided in facilitating the treatment decision making in clinical practice. Last but not least, LACA patients with obvious RD diagnosed from gynecological examination and MRI, who certainly benefited from completion hysterectomy after CCRT, were excluded from the current study, which further reduced the selection bias and strengthened the clinical applicability of the result for patients without obvious RD, who theoretically should be the most hesitant population to choose completion hysterectomy in daily practice. However, several limitations should also be noted. First, selection bias was hard to avoid given the retrospective nature of this study. While PSM cannot fully eliminate unmeasured confounding, it was indeed an option for reducing the selection bias. Second, the presence of UI was diagnosed via MRI scanning in the current study, which was essentially less trustworthy than a pathological diagnosis. Despite this, MRI might be the optimal tool we could choose to conduct the present study, given that surgery was not the first choice for patients with LACC. Third, the small sample size of patients with pathological RD≥1/2MI might mitigate the credibility of adding postoperative chemotherapy for these patients. Lastly, it was important to balance the risk and benefit from surgery after CCRT for LACA patients; however, the specific cost of treatment for every patient was hard to retrieve in the current study, which demanded further cost-effectiveness analysis in the future.

### Implications for practice and future research

Completion hysterectomy after CCRT for LACA patients remains a topic worth debating. We demonstrated that surgery could significantly increase the DMFS for LACA patients after definitive CCRT, while this benefit could not be conferred to the OS benefit. Nevertheless, the presence of UI served as a definitive indicator for receiving surgery after CCRT. Besides, patients with pathological RD≥1/2MI might benefit from postoperative chemotherapy, while patients without RD or with pathological RD<1/2MI did not. AC would always be a subset calling for innovative treatment in the future, and a more precise indication, combining molecular mutational traits for additional treatment would be needed.

## Supplementary Material

## Data Availability

All data that support the findings of this study are available from the corresponding author upon reasonable request.

## Research registration unique identifying number (UIN)

We registered the current study in http://clinicaltrials.gov, and a registration number of NCT06741046 was assigned.
