# Supplementary material for: Appraisal the significance of completion hysterectomy after definitive concurrent chemoradiotherapy for patients with locally advanced cervical adenocarcinoma: the ATTRACT study
Source: Int J Surg. 2025 Nov 5;112(1):1274–83. doi: 10.1097/JS9.0000000000003549 (PMC12825669; doi:10.1097/JS9.0000000000003549)
Supplement: Supplementary file 1 [file js9-112-1274-001.docx]

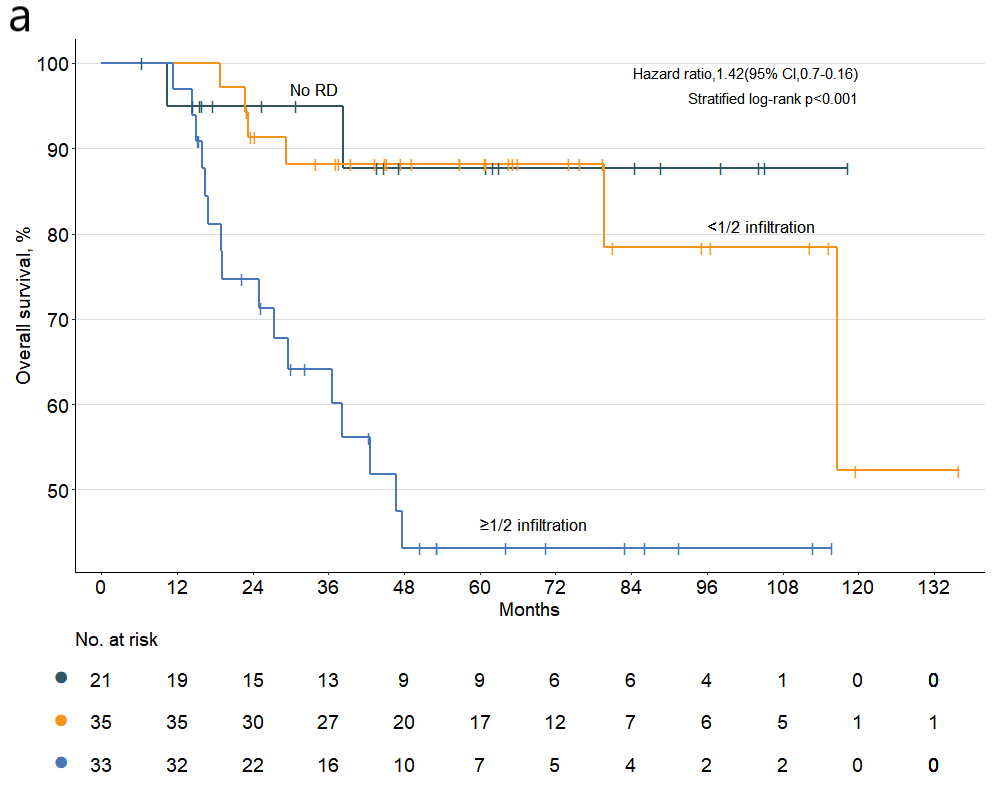

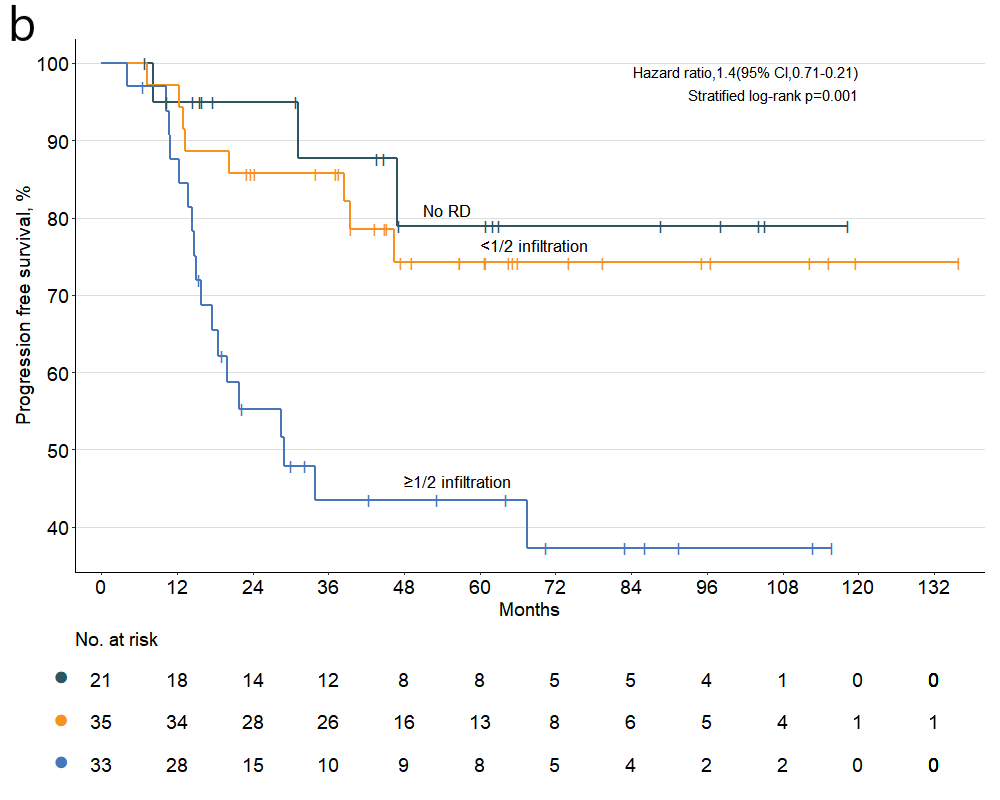


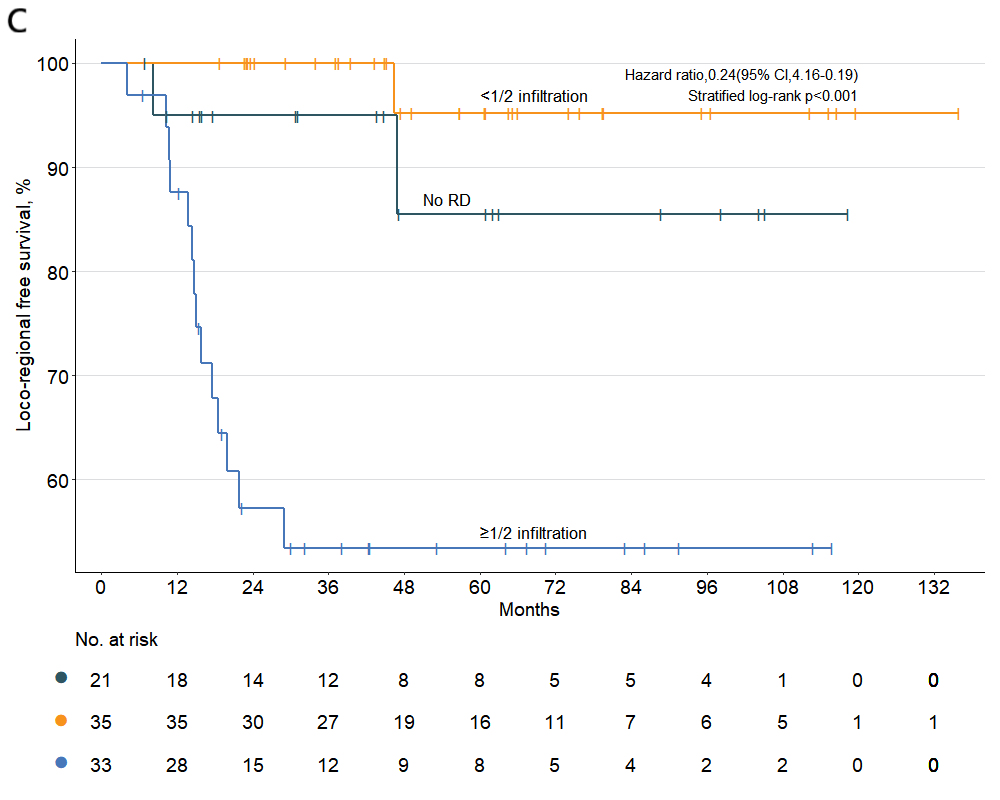

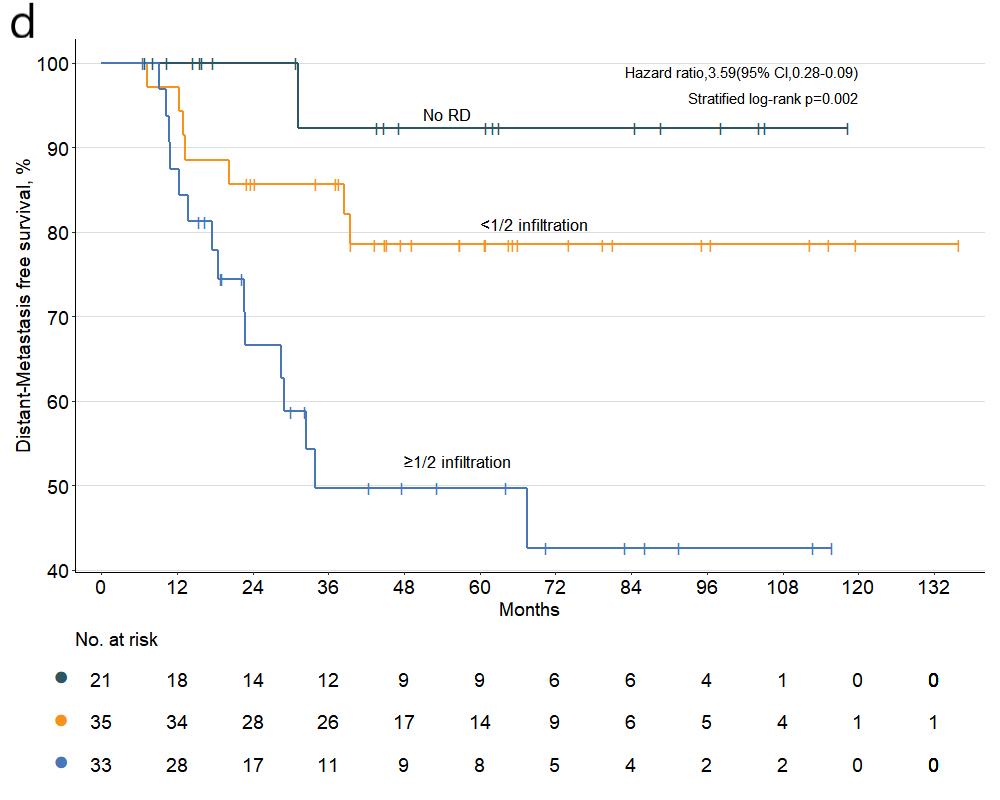


**Fig.S1** The comparison of OS (a), PFS (b), LRFS (c) and DMFS (d) between No residual disease, with residual disease<1/2 infiltration and with residual disease≥1/2 infiltration group among patients receiving surgery after chemoradiotherapy.


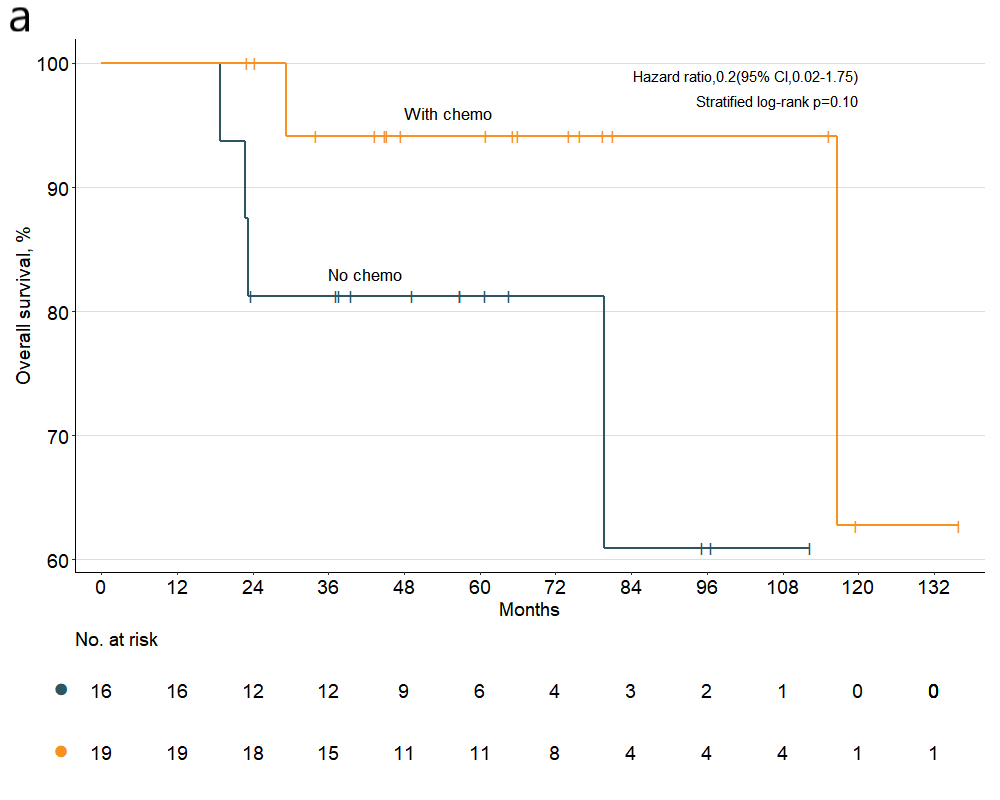

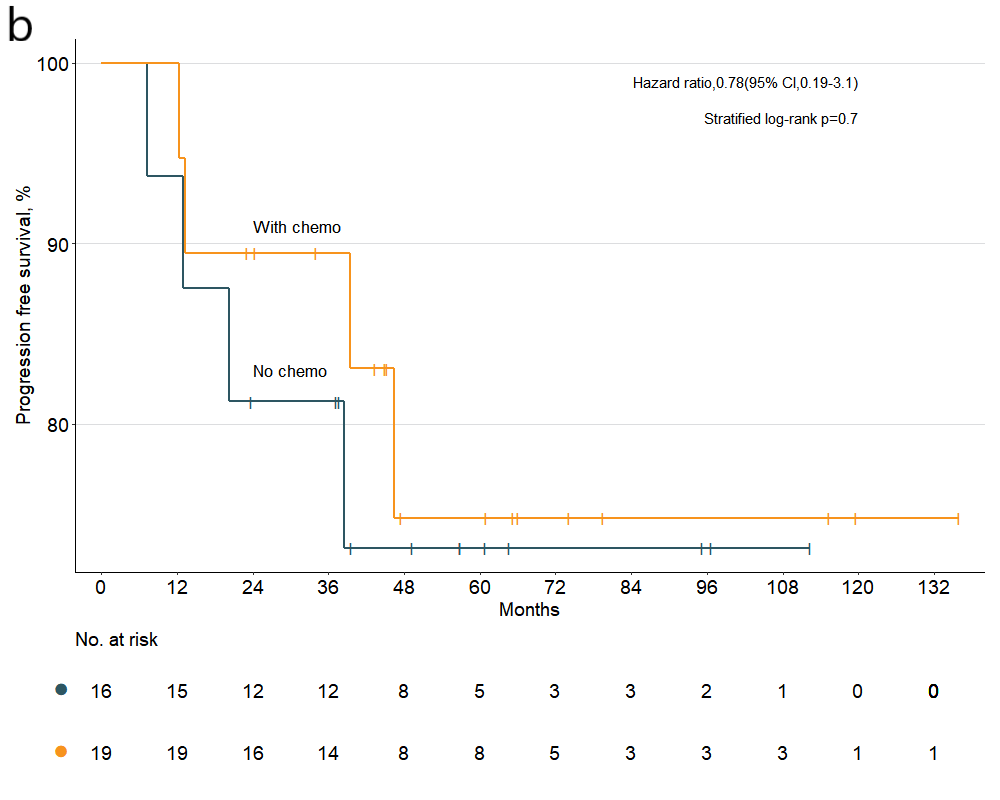

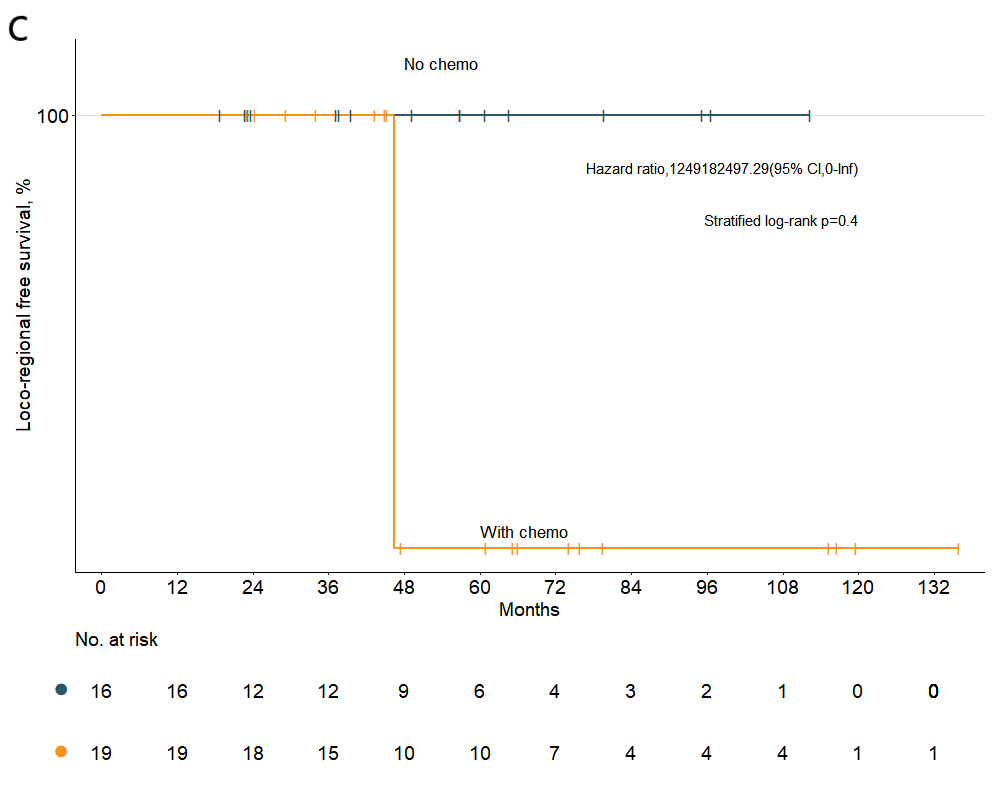

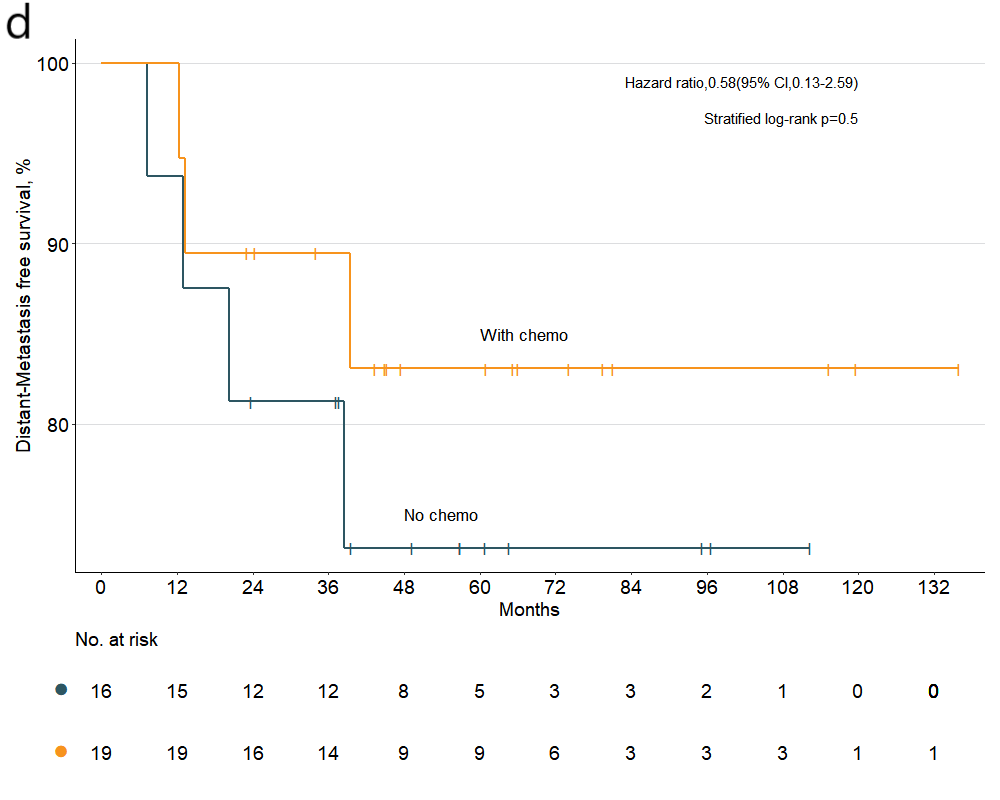


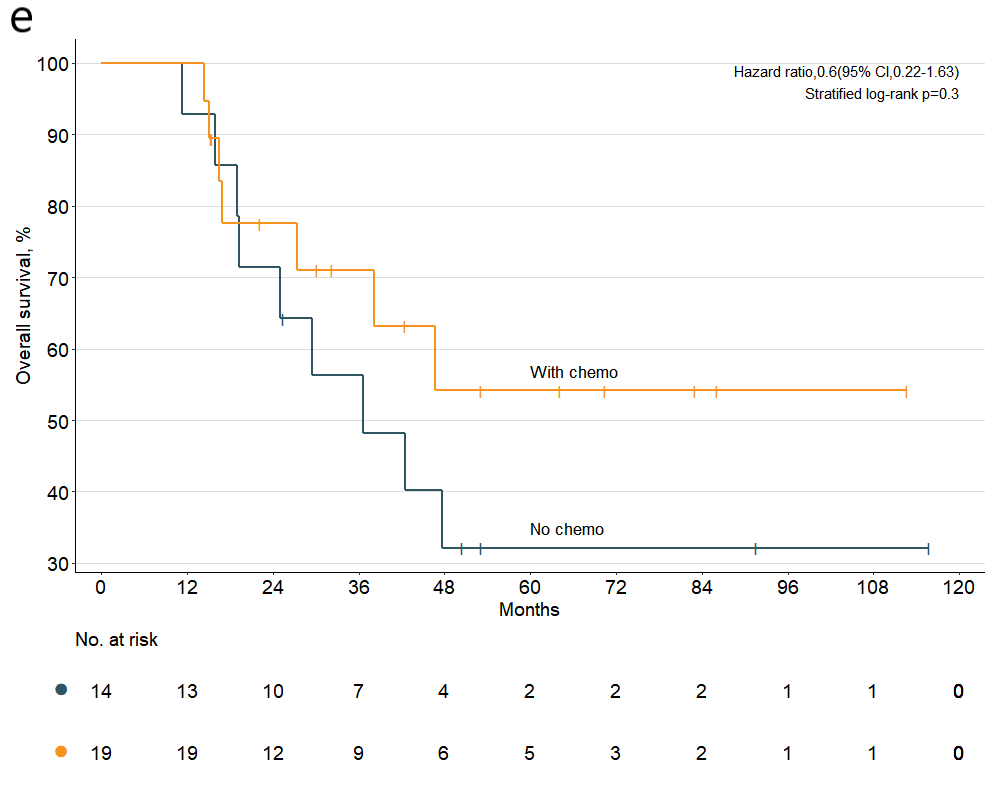

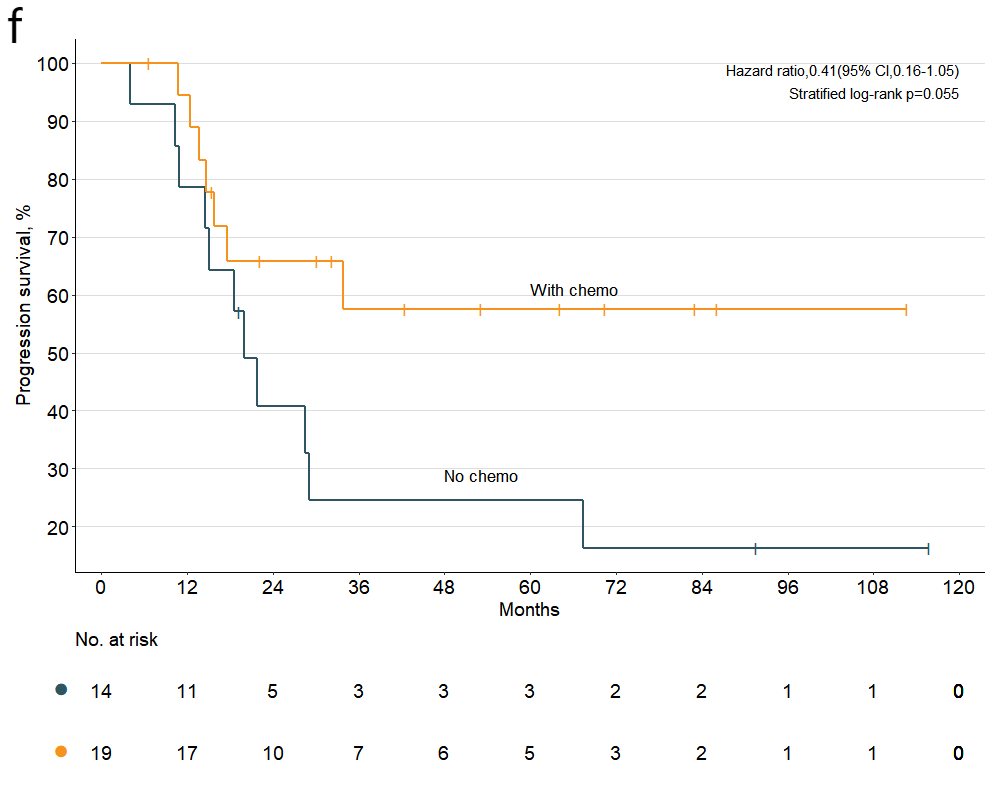

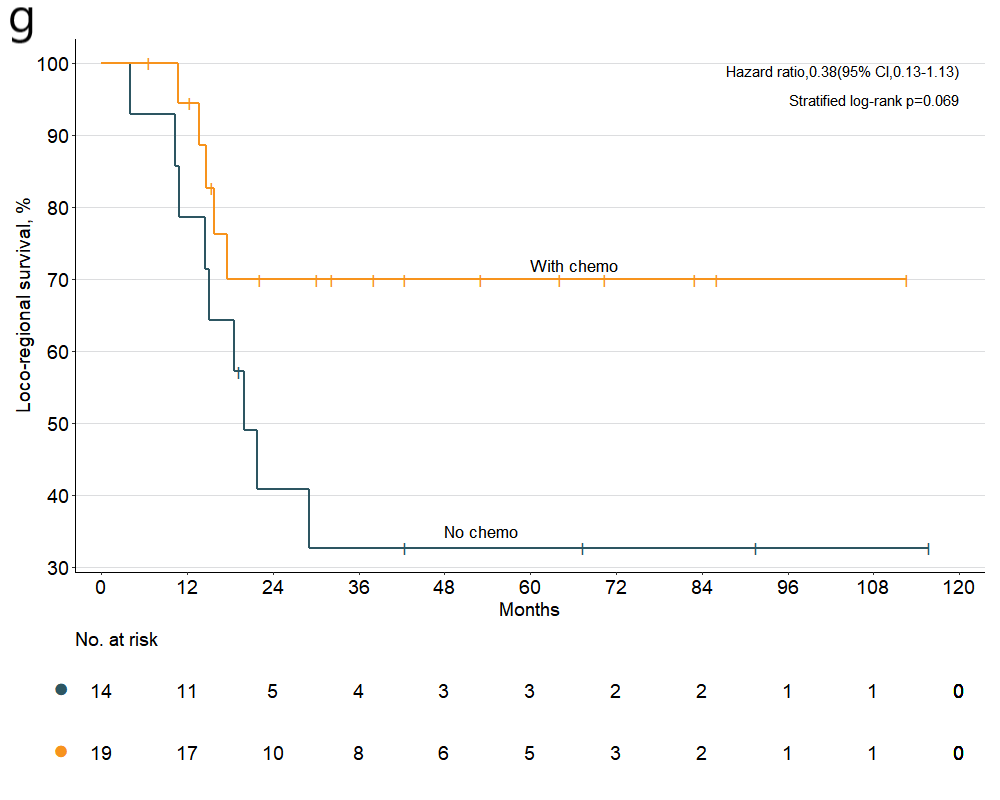

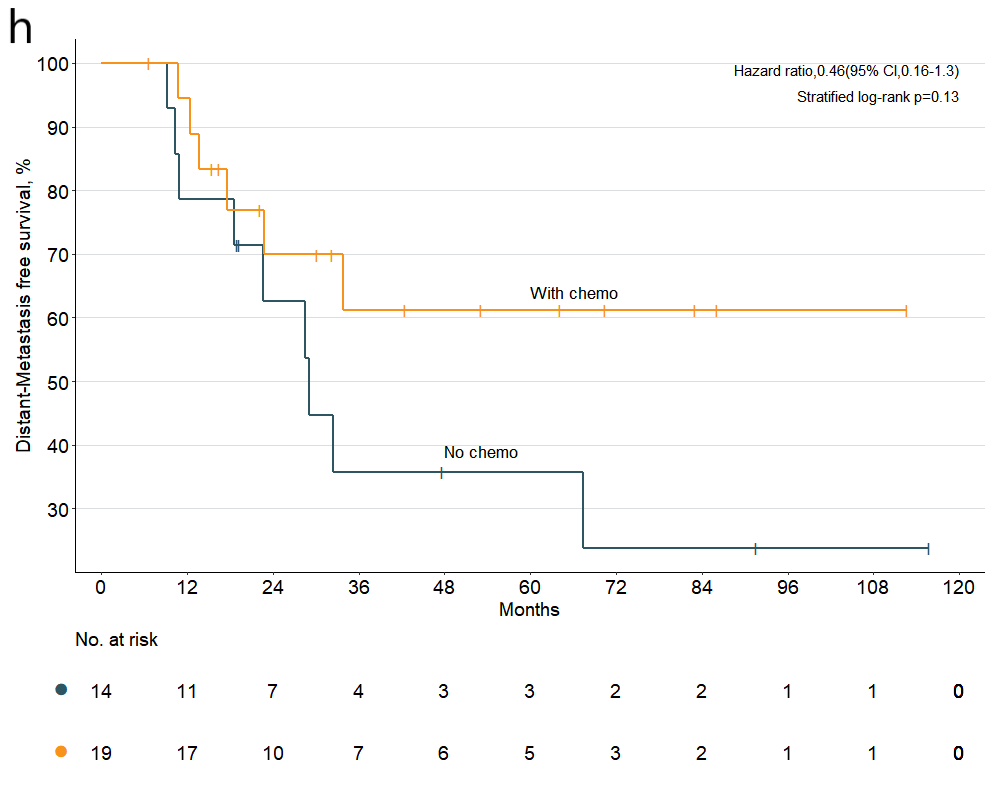


**Fig.S2** The comparison of OS (a), PFS (b), LRFS (c) and DMFS (d) between with post-operative chemotherapy and without post-operative chemotherapy group among patients with residual disease<1/2 infiltration. The comparison of OS (a), PFS (b), LRFS (c) and DMFS (d) between with post-operative chemotherapy and without post-operative chemotherapy group among patients residual disease≥1/2 infiltration.

| **Table.S1** The statistical power in certain subgroup analysis with limited sample size. | | | | |
| --- | --- | --- | --- | --- |
| **Comparison Category** | **OS** | **PFS** | **LRFS** | **DMFS** |
| **CCRT+Surgery VS CCRT alone after PSM** | 0.217 | 0.479 | 0.588 | 0.422 |
| **With chemo VS No chemo in patients with RD<1/2MI** | 0.207 | 0.585 | 0.477 | 0.602 |
| **With chemo VS No chemo in patients with RD≥1/2MI** | 0.312 | 0.599 | 0.611 | 0.546 |

CCRT: concurrent chemoradiotherapy; OS: overall survival; PFS: progression free survival; LRFS: loco-regional free survival; DMFS: distant metastasis free survival; RD: residual disease; MI: myometrial infiltration.
